# Enhancement of the Tolerogenic Phenotype in the Liver by ImmTOR Nanoparticles

**DOI:** 10.3389/fimmu.2021.637469

**Published:** 2021-05-25

**Authors:** Petr O. Ilyinskii, Christopher J. Roy, Julie LePrevost, Gina L. Rizzo, Takashi Kei Kishimoto

**Affiliations:** Selecta Biosciences, Watertown, MA, United States

**Keywords:** immune tolerance, ImmTOR, rapamycin, liver, LSECs, double-negative T cells, regulatory T cells

## Abstract

ImmTOR biodegradable nanoparticles encapsulating rapamycin have been shown to induce a durable tolerogenic immune response to co-administered biologics and gene therapy vectors. Prior mechanism of action studies have demonstrated selective biodistribution of ImmTOR to the spleen and liver following intravenous (IV) administration. In the spleen, ImmTOR has been shown to induce tolerogenic dendritic cells and antigen-specific regulatory T cells and inhibit antigen-specific B cell activation. Splenectomy of mice resulted in partial but incomplete abrogation of the tolerogenic immune response induced by ImmTOR. Here we investigated the ability of ImmTOR to enhance the tolerogenic environment in the liver. All the major resident populations of liver cells, including liver sinusoidal endothelial cells (LSECs), Kupffer cells (KC), stellate cells (SC), and hepatocytes, actively took up fluorescent-labeled ImmTOR particles, which resulted in downregulation of MHC class II and co-stimulatory molecules and upregulation of the PD-L1 checkpoint molecule. The LSEC, known to play an important role in hepatic tolerance induction, emerged as a key target cell for ImmTOR. LSEC isolated from ImmTOR treated mice inhibited antigen-specific activation of ovalbumin-specific OT-II T cells. The tolerogenic environment led to a multi-pronged modulation of hepatic T cell populations, resulting in an increase in T cells with a regulatory phenotype, upregulation of PD-1 on CD4^+^ and CD8^+^ T cells, and the emergence of a large population of CD4^–^CD8^–^ (double negative) T cells. ImmTOR treatment protected mice in a concanavalin A-induced model of acute hepatitis, as evidenced by reduced production of inflammatory cytokines, infiltrate of activated leukocytes, and tissue necrosis. Modulation of T cell phenotype was seen to a lesser extent after administration by empty nanoparticles, but not free rapamycin. The upregulation of PD-1, but not the appearance of double negative T cells, was inhibited by antibodies against PD-L1 or CTLA-4. These results suggest that the liver may contribute to the tolerogenic properties of ImmTOR treatment.

## Introduction

ImmTOR nanoparticles (formerly called SVP-Rapamycin) are comprised of rapamycin, an inhibitor of the mTOR pathway, embedded in a matrix of biodegradable poly(lactic acid) (PLA) polymer (reviewed in 1). ImmTOR has been shown to induce durable and antigen-specific tolerance in a variety of applications including mitigating immune responses against biological therapeutics (2–5), hepatotropic AAV gene therapy vectors (6), and autoantigens (7, 8). The principal target organs of biodistribution for intravenously delivered ImmTOR are spleen and liver (7). This is consistent with another published report that shows nanoparticle accumulation and capture by hepatic and splenic sinusoids (9). The liver is known to behave as a tolerogenic organ (10). Under normal conditions, there is an active suppression of immunity to a continuous flow of gut flora and food-borne antigens, which enter the liver *via* portal vessels to liver sinusoids (11). This process is essential to prevent unwanted immune stimulation to otherwise harmless digestive antigens and commensal bacterial antigens. The tolerogenic potential of the liver was first shown over 50 years ago, with an observation that MHC mismatched pigs could tolerate allogeneic liver transplants without immunosuppressive drugs. Moreover, porcine recipients of liver allografts were also capable of accepting other organ grafts, which normally would have been rejected in the absence of the liver allograft (12). Similarly, immune responses against the transgene product of AAV gene therapy expressed in the muscle can be mitigated by co-expression of the transgene in the liver (13).

Despite the propensity towards tolerogenic immune responses in the liver, robust effector immune responses can be mounted in the liver in the case of liver-tropic viral infections and liver-targeted autoimmune diseases. In humans, immunosuppressive drugs must be used in liver transplantation, although up to 20% of patients can be gradually weaned from these drugs over time while maintaining graft function (14). The liver contains several unique cell populations capable of presenting antigens, such as Kupffer cells (KC), the most abundant liver resident macrophage population possessing scavenger/phagocytic function (15), and liver sinusoidal endothelial cells (LSECs), the most abundant non-parenchymal hepatic cell population which line the sinusoidal capillary channels and are involved in filtering blood passing through the liver. LSECs have high endocytic capacity and are capable of presenting antigen to T cells (16). The balance between tolerogenic immune responses and effector immune responses is likely influenced by the phenotype of antigen-presenting cells in the liver, such as expression of co-stimulatory molecules, CD80 and CD86, which promote effector immune responses, and checkpoint molecules, such as PD-L1, which promote tolerogenic immune responses.

In this study we followed trafficking of fluorescent-labeled ImmTOR particles to the liver, showing its simultaneous uptake by all major liver cell populations. ImmTOR induced a prolonged tolerogenic phenotype in KCs and LSECs, characterized by down-regulation of MHC class II and co-stimulatory molecule expression and profound upregulation of PD-L1. This, in turn, led to induction of major and persistent changes in hepatic T cell populations, with an overall decrease in CD4 and CD8 T cells, a marked increase in PD-1 expression, and induction of T cells with a regulatory phenotype (CD25^+^, CD127^low^, PD-1^+^). Additionally, the emergence of a large population of double-negative (CD4^-^, CD8^-^) T cells was observed in the liver, but not the spleen. The upregulation of PD-1, but not the increase in double negative T cells, was partially dependent on the PD-L1/PD-1 axis and on CTLA-4. Collectively, upon the exposure to ImmTOR, most of hepatic T cells acquired an immunosuppressive or anergic phenotype, which was maintained for at least 2 weeks after a single treatment. ImmTOR treatment also protected mice in a concanavalin A-induced model of acute hepatitis.

## Materials and Methods

### ImmTOR and Other Nanoparticles

Rapamycin containing nanoparticles (ImmTOR) were manufactured as described earlier (2, 7). Briefly, PLA, pegylated polylactic acid (PLA-PEG), and rapamycin were dissolved in dichloromethane to form the oil phase. An aqueous solution was then added to the oil phase and emulsified by sonication (Branson Digital Sonifier 250A). Following emulsification, a double emulsion was created by adding an aqueous solution of polyvinylalcohol and sonicating a second time. The double emulsion was added to a beaker containing phosphate buffer solution and stirred at room temperature for 2 h to allow the dichloromethane to evaporate. The resulting NPs were washed twice by centrifuging at 75,600 × g and 4°C followed by resuspension of the pellet in PBS. Fluorescent Cy5-containing NPs were manufactured as described above using PLA-Cy5 conjugate. PLA with a butyl amine end group was prepared from PLA-acid, which was then treated with Cy5-acid in the presence of a coupling agent (O-(Benzotriazol-1-yl)-N,N,N′,N′-tetramethyluronium tetrafluoroborate) to afford the conjugates. ImmTOR doses were based on rapamycin content ranging from 200 to 400 µg. Empty nanoparticles were manufactured in an identical manner, but without rapamycin. Rapamycin (sirolimus) was manufactured by Concord Biotech (Ahmedabad, India).

### Mice

Immunologically naïve, female C57BL/6 mice aged 36-52 days (or 17-18g) were purchased from Charles River Laboratories (Wilmington, MA). Similarly aged B6.Cg-Tg(TcraTcrb)425Cbn/J mice (also known as OT-II mice), expressing the T cell receptor (TCR), which is specific for chicken ovalbumin 323-339 peptide (OVA_323-339_ or OP-II) in the context of I-A^b^ resulting in CD4^+^ T cells that primarily recognize OP-II when presented by the MHC-II molecule were purchased from Jackson Laboratories (Bar Harbor, ME). To minimize the potential effects of stress, mice were acclimated to the Animal Care Facility at Selecta for at least three days prior to injection. All the experiments were conducted in strict compliance with NIH Guide for the Care and Use of Laboratory Animals and other federal, state and local regulations and were approved by Selecta’s IACUC.

### Animal Injections

Mice were injected (i.v., tail vein or retro-orbital plexus) with ImmTOR or empty nanoparticles in the effective range of 200-400 µg. Molar equivalent of soluble rapamycin was administered i.p.

### Sample Collection and Flow Cytometry

For given timepoints (most of the time, several overlapping time-points were tested using the same set of treatments, always including naïve and/or placebo or free rapamycin controls), mice were euthanized, livers and/or spleens harvested and rendered into single cell suspensions *via* collagenase 4 (Worthington Biochemical, Lakewood, NJ) enzymatic digest according to manufacturer’s recommended protocol. Next, a red blood cell lysis step was performed for 5 min at room temperature in 150 mM NH_4_Cl, 10 mM KHCO_3_, 10 μM Na_2_-EDTA; washed in PBS, 2% bovine serum; then filtered on a 70 µm nylon mesh. LSECs were isolated *via* CD146 positive selection with immunomagnetic beads according to manufacturer’s instructions (Miltenyi Biotec, San Diego, CA). To prevent non-specific antibody binding, cells were incubated 20 min. on ice with anti-CD16/32 then stained with antibodies (all from BioLegend, San Diego, CA) for given cell surface phenotype. Analysis was performed *via* FACSCanto flow cytometer (BD Biosciences) with subsequent data analysis using FlowJo software (TreeStar, Ashland, OR).

### Characterization of Liver Cell Subpopulations

Flow cytometry was used for the phenotyping of hepatocytes, LSEC, KC, hepatic stellate cells (HSC) and liver T cells from liver cell suspension and of LSEC post CD146 positive selection. Dead cells were always excluded from analysis. Phenotypic changes were assessed as percentage of parent population as shown; measuring of absolute mean fluorescent intensity (MFI) gave essentially the same results. Gating strategies for all the major hepatic cell populations assessed in the study are shown in **Supplementary Figure S1**. All antibodies to cell surface markers were from BioLegend (San Diego, CA), with the exception of that against LRP-1 (A2MR-α2) being from Thermo Fischer (Waltham, MA). The following primary antibodies were used to identify liver parenchyma cells: anti-LRP-1 (conjugated with R-PE using SiteClick R-PE labeling kit (Thermo Fischer) according to manufacturer’s instructions), F4/80 (BV510), anti-CD68 (APC/Cy™7), anti-CD11b (PE/Cy7), anti-mannose receptor (MR) (BV-eFlour450), anti-CD146-FITC, anti-CD38 (APC/Cy7) and anti-GFAP (BV421). To confirm the purity of the LSEC population after CD146 positive selection anti-MR (BV eFlour450), anti-F4/80 (BV 510) and anti-CD68 (APC/Cy7) were used. For LSEC and KC phenotyping, the following antibodies were utilized: anti-MHCII (Alexa Flour^®^ 488), anti-CD80 (PE), anti-CD86 (PE/Cy7) and anti-PD-L1 (PerCP-Cy5.5), all from BioLegend. The following antibodies were used for T cell differentiation and characterization; anti-CD127 (PE), anti-PD-1 (PerCP-Cy5.5), anti-CD4 (PE/Cy7), anti-CD25 (APC/Cy7), anti-CD3 (BV421), anti-CD8α (BV510), anti-CD62L (Alexa Flour^®^ 488), anti-CD44 (PE) and anti-NK1.1 (APC-Fire). Annexin B was used to evaluate cell apoptosis. Additionally, anti-CD11c (BV510), anti-PDCA1 (Alexa Flour^®^ 488), anti-CD45 (APC/Cy7), anti-CD152(PE/Cy5) and anti-MHCI (PerCP-Cy5.5) were used to analyze dendritic cells (DC) and their activation status. Cells were incubated with anti-CD16/32 antibodies to prevent non-specific binding, then incubated with primary antibodies for 30 min at 4°C and washed. Analysis of cells were carried out on BD FACSCanto™ II flow cytometer (BD Biosciences) with data analysis using FlowJo software (TreeStar, Ashland, OR).

### Cell Proliferation and Cytokine Secretion *In Vitro*

LSEC were purified as described above and KC were purified using positive selection F4/80 microbeads (Miltenyi Biotec) with >80% purity of either population confirmed by flow cytometry. For cell proliferation studies LSEC isolated from ImmTOR-treated or naïve mice were cultured in limiting dilutions (starting at 1.25 x 10^5^ cells/well) with a fixed number of OT-II splenocytes (2.5 x 10^4^ cells/well) stimulated with OVA_323-339_ peptide (Anaspec, Fremont, CA) at 1 µg/ml. Cultures were carried out in triplicates in 96-well round-bottomed plates and cell proliferation was evaluated 72 hours after initiating the cultures using two separate methods, namely resazurin-based fluorescence (17) with PrestoBlue™ HS cell viability reagent (Thermo Fisher, Waltham, MA) according to manufacturer’s instructions and *via* intracellular flow cytometry using labeling with anti-Ki-67 (Alexa Fluor^®^ 647, BioLegend), a protein known to be absent in non-dividing cells (18) and to positively correlate with mouse T cell proliferation (19).

Serum cytokine levels were analyzed with Meso Scale Discovery (MSD) 96-Well MULTI-SPOT^®^ Ultra-Sensitive Human Immunoassay Kits, using electrochemiluminescence detection on an MESO^®^ QuickPlex SQ 120 with Discovery Workbench software (version 3.0.18) (MSD^®^, Gaitherburg, MD). Cytokines were measured using the U-PLEX TH1/TH2 combo (ms) 10-plex kit and the U-PLEX TGF-β Combo (ms) 3-plex kit. Assays were performed according to manufacturer’s instructions, and without alterations to the recommended standard curve dilutions. OR).

### Concanavalin A Challenge Model

Concanavalin A (Con A) induced liver toxicity model was employed essentially as earlier described (20, 21). Briefly, mice were injected (i.v., r.o.) Con A at 12 mg/kg and then terminally bled at 6, 8, 12 or 24 hours post-challenge with cytokine levels in serum determined by MSD as described above and liver tissues collected simultaneously for single-cell suspension analysis by flow cytometry as described above or for hematoxylin-eosin staining followed by microscopic evaluation.

### Statistical Analysis

Statistical analyses were performed using GraphPad Prism 8.0.2. To compare the mouse experimental groups pairwise either multiple t test (for several time-points) or Mann-Whitney two-tailed test (for a single time-point; individual comparison of two groups presented within the same graph) were used. Significance is shown for each figure legend (* – p < 0.05, ** – p < 0.01; *** – p < 0.001; **** – p < 0.0001; not significant – p > 0.05). All data for individual experimental groups is presented as mean ± SD (error bars).

## Results

### ImmTOR Trafficking to Liver Cell Populations

**Figure 1:**
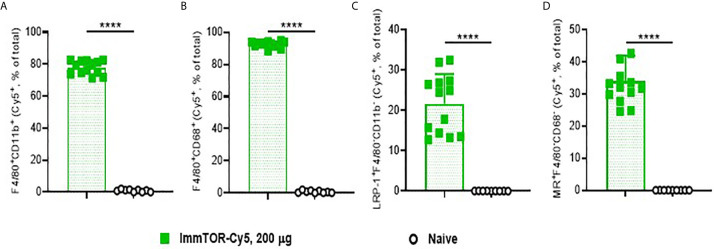
ImmTOR traffics to major liver cell populations after intravenous inoculation. Cy5-labeled ImmTOR particles (200 µg) were administered into the venous circulation *via* the retro-orbital venous sinus. Livers were harvested at day 7 and processed to single-cell suspensions, which were stained with antibodies to markers indicated and analyzed by flow cytometry. Fractions of Cy5-positive cytokine-producing **(A)** and phagocytic **(B)** Kupffer cells (F4/80^+^CD11b^+^ and F4/80^+^CD68^+^, respectively) as well as of hepatocytes **(C)** (LRP-1^+^F4/80^–^CD11b^–^) and LSEC **(D)** (MR^+^F4/80^–^CD68^–^) are shown (% of total). Summaries of three independent experiments in which identical time-points were assessed are shown (n = 9-13 mice/group). Background fluorescence in naïve mice is also shown. Statistical difference in the size Cy5-positive fractions *vs.* that in naïve mice is shown (**** – p<0.0001; Mann-Whitney test).

ImmTOR has been previously shown to preferentially accumulate in the liver and spleen after intravenous delivery (7). In order to discern its hepatic trafficking in more detail, we analyzed individual liver cell populations by flow cytometry after injection of fluorescent-labeled ImmTOR (**Figure 1**). Not surprisingly, there was a massive uptake of ImmTOR by Kupffer cells, especially those with a phagocytic phenotype (**Figures 1A, B**). Moreover, there was significant ImmTOR uptake by hepatocytes (**Figure 1C**) and LSECs (**Figure 1D**). Similar results were seen at earlier and later time-points, spanning from one day to two weeks post injection (not shown). Collectively, intravenous injection of ImmTOR led to its simultaneous uptake by all major resident liver cell populations tested.

### Induction of a Tolerogenic Profile in Professional and Non-Professional Liver APC

**Figure 2:**
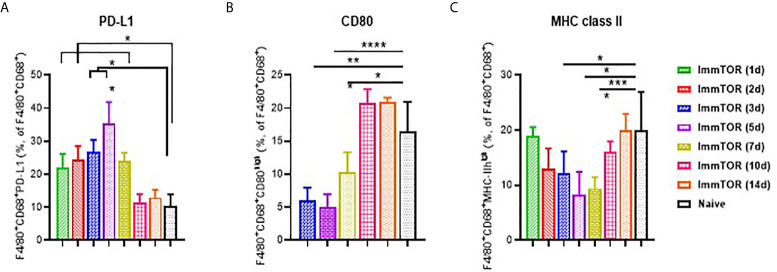
Induction of a tolerogenic phenotype in Kupffer Cells (KC) by ImmTOR. KC were stained in liver cell suspensions following treatment of mice with 200 µg ImmTOR (i. v.) Fractions of PD-L1+ **(A)**, CD80+ **(B)** and MHC-II+ **(C)** phagocytic Kupffer cells (KC, identified as F4/80+CD68+) are shown. Summaries of four independent experiments in which different and overlapping time-points were assessed are shown (n = 3-12 mice/group). Statistical difference in the size of respective fractions at different time-points *vs.* that in naïve mice is shown (* – p<0.05, ** – p<0.01, *** – p<0.001, **** – p<0.0001; Mann-Whitney test).

Expression of cell surface molecules on phagocytic KCs that play key roles in antigen presentation and immune co-stimulation were affected as early as 1-3 days after ImmTOR treatment (**Figure 2**). Specifically, expression of the immune checkpoint ligand, PD-L1, was already elevated at day 1 (**Figure 2A**), while expression of MHC class II and the co-stimulatory CD80 molecule were decreased by day 3 (**Figures 2B, C**). All of these effects peaked around days 5-7 post-ImmTOR administration and returned to baseline levels by day 10. Other populations of professional APC, such as myeloid DC, showed similar increases in PD-L1 and decreases in CD80 expression, while plasmacytoid DC and cytokine-producing KC showed modest but significant decreases in CD80 and CD86 expression (**Supplementary Figure S2**).

**Figure 3:**
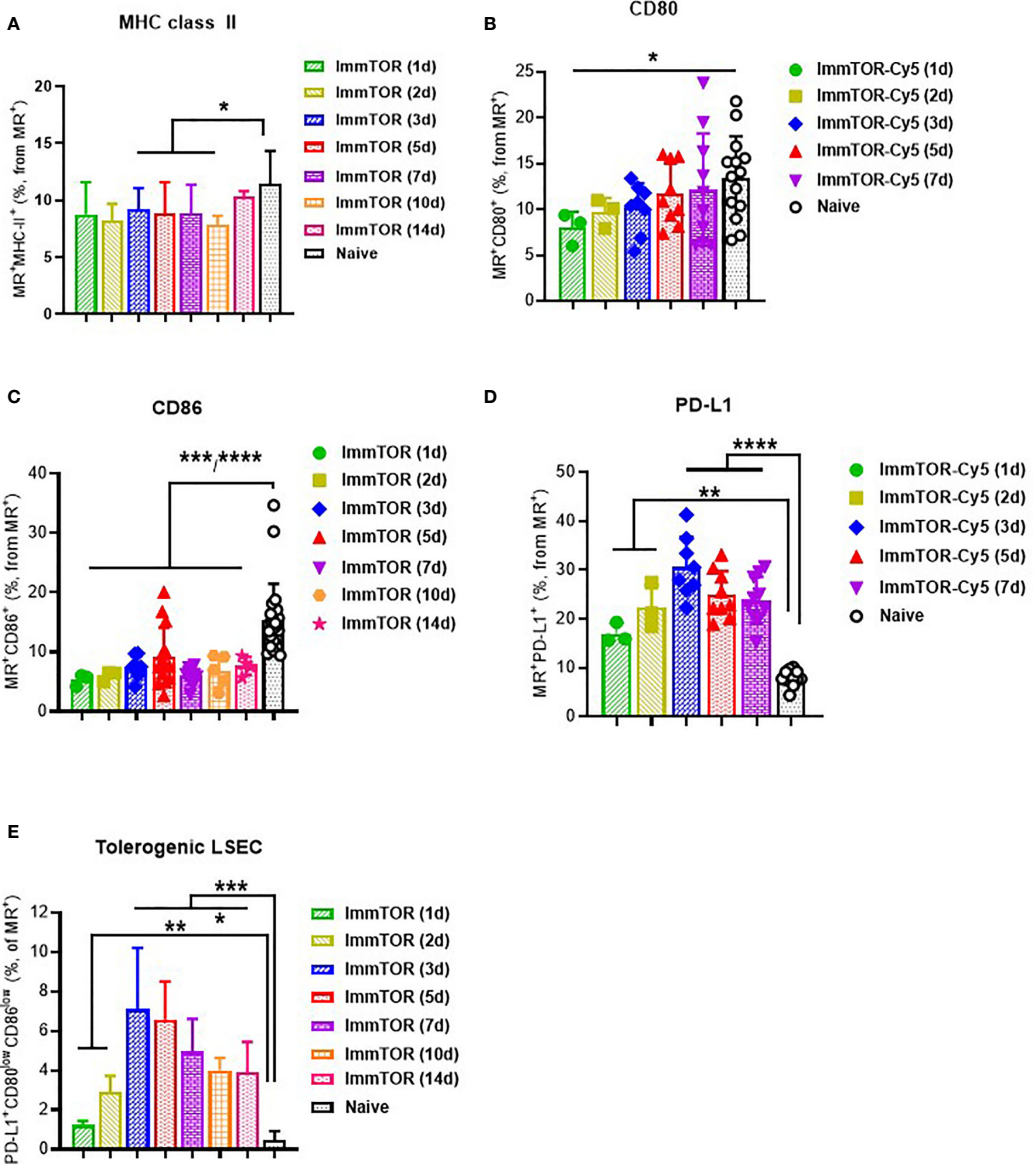
ImmTOR induces a tolerogenic phenotype in LSECs. LSEC were isolated from liver at different time-points after i.v. injection of ImmTOR at 200 µg and analyzed by flow cytometry. Fractions of MHC-II+ **(A)**, CD80+ **(B)**, CD86+ **(C)**, and PD-L1+ **(D)** liver sinusoid endothelial cells (LSEC, identified as MR+F4/80–CD68–) are shown. The fraction of tolerogenic LSEC (identified as PD-L1+CD80lowCD86low) is also shown **(E)**. Summaries of five independent experiments are shown in which different and overlapping time-points were assessed (n=3-20 mice per group). Statistical difference in the size of respective fractions at different time-points *vs.* that in naïve mice is shown (* – p<0.05, ** – p<0.01, *** – p<0.001, **** – p<0.0001; Mann-Whitney test).

A broad effect of ImmTOR was detected when analyzing the surface molecule expression profile of LSECs, which have been shown to play a major role in tolerogenic immune responses in the liver (16). Specifically, a consistent suppression of MHC class II, CD80 and especially, CD86 was detected as early as one day after ImmTOR treatment and then maintained throughout the first week after ImmTOR administration (**Figures 3A–C**). Of these, CD80 was downregulated early, but then gradually increased over the first week (**Figure 3B**), while MHC-II was modestly downregulated over days 1-10 post injection (**Figure 3A**). CD86 expression was profoundly suppressed for at least two weeks after ImmTOR treatment (**Figure 3C**). None of these effects were observed if placebo nanoparticles (NP-Empty) were used (not shown).

In contrast, expression of PD-L1 was markedly elevated on LSECs at 1 day after ImmTOR administration and peaking around days 3-5 post-treatment (**Figure 3D**). When combined with analysis of CD80/86 expression, a profound increase in LSEC with a tolerogenic phenotype (PD-L1^+^CD80^low^CD86^low^) was apparent during the first week after ImmTOR administration, which was maintained through at least day 14 (**Figure 3E**). By this time CD80 and MHC class II (**Figures 3A, B**) expression was gradually restored to baseline levels, while CD86 expression remained suppressed (**Figure 3C**). A similar phenotype of LSEC was observed if a purified LSEC population selected for CD146 expression was used (not shown) and this tolerogenic LSEC surface phenotype was the same irrespective of whether MR or CD146 was used for LSEC identification (**Supplementary Figure S3**). As with professional hepatic APC, no effect on LSEC surface expression of CD80, CD86 and MHC class II molecules was seen when NP-Empty was used (see **Supplementary Figure S3** for representative images).

There was little or no difference in expression of PD-L1 or MHC class II when comparing total hepatocytes from mice treated with ImmTOR *vs* naïve controls (**Supplementary Figure S4**). However, those hepatocytes that took up ImmTOR, as evidenced by use of fluorescent-labeled ImmTOR, showed a profound upregulation of PD-L1 and down-regulation of MHC class II expression. Specifically, such fluorescent ImmTOR-positive hepatocytes expressed two times less MHC class II (**Supplementary Figure S4A**) and two times more PD-L1 (**Supplementary Figure S4B**) than ImmTOR-negative hepatocytes or hepatocytes from naïve untreated mice. A similar pattern was observed in hepatic stellate cells (**Supplementary Figure S5**), which by day 5 took up ImmTOR particles with similar efficiency as other parenchymal cells (**Supplementary Figure S5A**) and then upregulated PD-L1 and downregulated MHC class II molecules (**Supplementary Figures S5B, C**), but only in those cells that took up ImmTOR (**Supplementary Figures S5D, E**). The same effect was seen at 7 days post-injection (not shown) with MHC class II expression on HSC decreased from being nearly 100% in HSC that were ImmTOR-negative to less than 20% of cells that were positive for fluorescent ImmTOR.

### Modulation of Hepatic T Cells by ImmTOR

**Figure 4:**
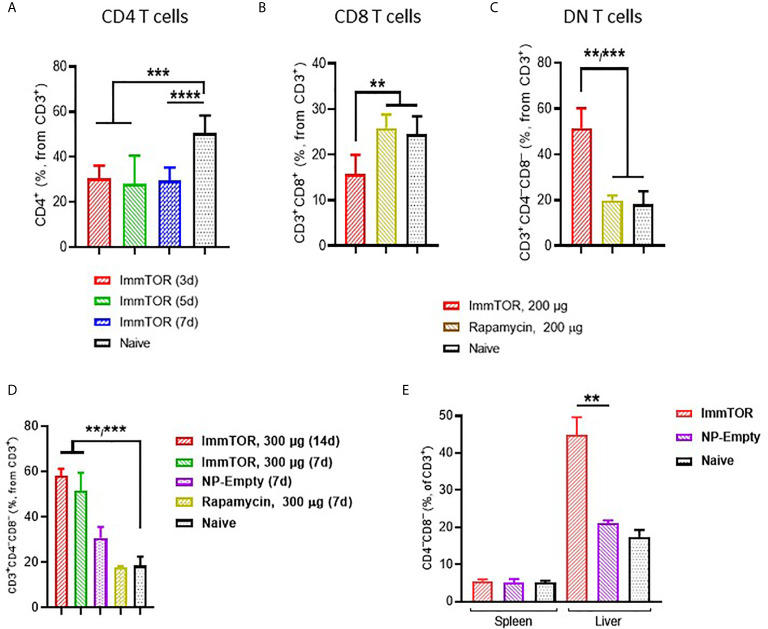
ImmTOR treatment leads to the emergence of double-negative T cells in the liver but not spleen. Livers and/or spleen were processed and analyzed after i.v. injection of ImmTOR at 200 **(A–C)** or 300 µg **(D, E)** or the same dose of free rapamycin or empty nanoparticles (NP-Empty). Fractions of CD4^+^ **(A)**, CD8^+^ **(B)** and double-negative or CD4^–^CD8^–^ **(C, D)** hepatic or hepatic and splenic **(E)** T cells (identified as CD3^+^ cells within lymphocyte gate) are shown. Timing of cell analysis is indicated in **(A, D)** analysis shown in other panels was done at either five **(E)** or seven **(B, C)** days post injection. Summaries of three **(A)** or two **(B, C)** independent experiments are shown (n=5-13 or 4-8 mice/group, respectively), or representative results of individual studies (n=3-6 mice/group) repeated at least twice **(D, E)**. Data shown in A result from analysis of different and overlapping time-points. Statistical difference in the size of respective fractions at different time-points *vs.* that in naïve mice or mice injected with free rapamycin or NP-Empty is shown (** – p<0.01, *** – p<0.001, **** – p<0.0001; Mann-Whitney test).

ImmTOR administration led to a profound impact on hepatic T cells (**Figure 4**). Specifically, a massive decrease in CD4^+^ T cells was observed as early as 3 days after ImmTOR treatment (**Figure 4A**), which was accompanied by a decrease of CD8^+^ cells as well (**Figure 4B**). There was no evidence of increased apoptosis among either CD4 or CD8 T cells whether 7-AAD (**Supplementary Figures S6A, B**) or Annexin B (not shown) were used. However, there was a substantial increase in double-negative (DN, CD3^+^CD4^–^CD8^–^) T cells that persisted for at least 14 days (**Figures 4C, D**). The increase in DN T cells was not reproduced by systemic injection of free rapamycin (**Figures 4B–D**), but was observed to a lesser degree with empty particles (NP-Empty) (**Figure 4D**). This effect was observed only in hepatic T cells, as a change in DN T cells was not seen in splenic T cells (**Figure 4E**).

**Figure 5:**
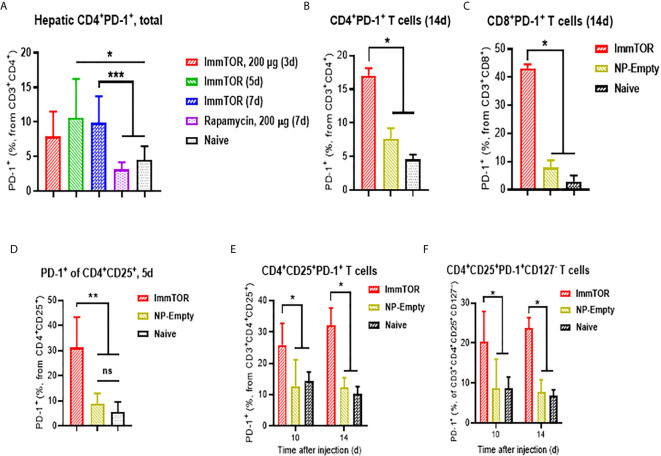
ImmTOR treatment leads to upregulation of PD-1 expression on hepatic T cells and regulatory T cells. T cells were directly stained in liver cell suspensions and analyzed after i.v. injection of ImmTOR at 200-400 µg or the same dose of free rapamycin or NP-Empty. Fractions of PD-1+ hepatic T helpers or CTL (identified as CD3^+^CD4^+^ or CD3^+^CD8^+^, correspondingly) is shown **(A–C)** as well as fractions of PD-1^+^ T effectors **(D, E)**, identified as CD3^+^CD4^+^CD25^+^) and ‘classic’ Tregs **(F)**, CD3^+^CD4^+^CD25^+^CD127^–^). Summaries of three **(A)** or two **(D)** independent experiments are shown (n=4-13 or 4-9 mice/group, respectively), or representative results of individual studies (n=3-5 mice/group) repeated at least twice **(B, C, E, F)**. Data shown in **(A)** result from analysis of different and overlapping time-points. Statistical difference in the size of respective fractions from ImmTOR-treated animals *vs.* that in naïve mice or those treated with NP-Empty or free rapamycin is shown (* – p<0.05, ** – p<0.01, *** – p<0.001; Mann-Whitney test). ns, not significant.

**Figure 6:**
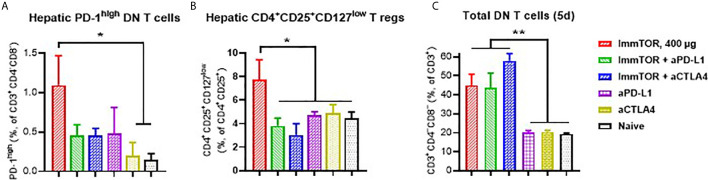
Effect of anti-PD-L1 and anti-CTLA-4 antibodies on ImmTOR-driven changes in hepatic T cells. Hepatic T cells were analyzed by flow cytometry 7 days after treatment with ImmTOR alone (400 µg), anti-PD-L1 or anti-CTLA-4 antibodies (i.p., 100 µg, three times with 3d intervals starting 3 days prior to ImmTOR injection), or ImmTOR combined with anti-PD-L1 or anti-CTLA-4 antibodies. Fractions of PD-1high DN T cells **(A)**, regulatory T cells **(B)**, and DN T cells **(C)**, are shown. Representative results of individual study repeated at least twice is shown (n=3 mice/group). Statistical difference in the size of respective fractions from ImmTOR-treated animals *vs.* other treatments is shown (* – p<0.05, ** – p<0.01; unpaired t-test).

Additionally, ImmTOR administration led to induction of PD-1 expression on remaining CD4^+^ and CD8^+^ T cell subpopulations within three days and maintained for at least 14 days post-administration and was not seen when equal doses of free rapamycin were used (**Figures 5A–C**). Empty nanoparticles showed a modest, non-significant trend to increasing PD-1 expression on T cells. The increase in PD-1 expressing T cells was more pronounced on CD4^+^CD25^+^ T cell cells (**Figures 5D, E**) and specifically on T cells with a regulatory phenotype (CD4^+^CD25^+^CD127^low^ (22, 23); (**Figure 5F**). However, there was no significant increase in intracellular FoxP3 expression within hepatic CD4^+^CD25^+^ cells (**Supplementary Figure S6C**). We then tested whether immune checkpoint-related pathways are involved in this process, especially taking into consideration ImmTOR-mediated PD-L1 elevation on hepatic professional and non-professional APC (**Figures 2**, **3**). ImmTOR-mediated increase in PD-1^high^ hepatic T cells (**Figure 6A**) and CD4^+^CD25^+^CD127^low^ T cells (**Figure 6B**), but not the emergence of hepatic DN T cells (**Figure 6C**), was inhibited by anti-PD-1 or anti-CTLA-4 antibodies.

There was no significant impact of ImmTOR on hepatic NK T cells (**Supplementary Figure S6D**) and the key features of T cell impact such as CD4 and CD8 surface downregulation, DN T cell emergence and PD-1 surface induction were the same irrespective of whether female (**Figure 5**) or male (**Supplementary Figure S7**) mice were used to assess ImmTOR effects. ImmTOR treatment did not affect surface expression of CTLA-4 and CD28 on hepatic T cells (not shown).

### ImmTOR-Mediated Suppression of Hepatic Immune Activation *In Vitro* and *In Vivo*

**Figure 7:**
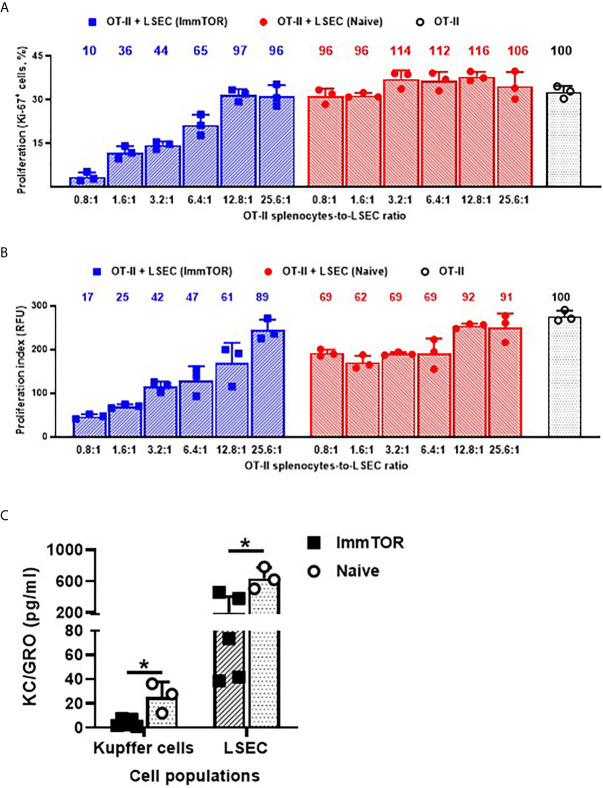
LSEC from ImmTOR-treated mice inhibit T cell proliferation *in vitro* and together with Kupffer cells, exhibit diminished KC/GRO secretion. LSEC were isolated from ImmTOR-treated (300 µg, 7 days) and intact animals and co-incubated at limiting dilutions with splenic derived OT-II cells and OVA_323-339_ (1 µg/ml). Cell proliferation index was measured by the percentage of Ki-67-positive cells **(A)** or by intensity of PrestoBlue fluorescent staining **(B)** as described in Materials and Methods with relative proliferation at each LSEC-to-splenocyte ratio also shown *vs.* positive (no LSEC) OT-II control as 100%. Experiment was performed twice with the same outcome and representative results are shown. **(C)** – LSEC and KC were purified from ImmTOR-treated (7 days) and intact animals, their purity confirmed by FACS and then were plated at 200,000 cells/well with cytokine concentration in supernatants assayed at 7 days. Levels of KC/GRO secretion are shown with statistical significance indicated (* – p<0.05; Mann-Whitney test).

The ability of ImmTOR to suppress immune activation was further assayed using several ex vivo and *in vivo* models. Firstly, LSEC from ImmTOR-treated or naïve mice were isolated and co-incubated with cognate-peptide stimulated splenocytes from OT-II mice. While LSEC from naïve mice exhibited minimal and non-specific inhibitory effect on OT-II cell proliferation, the effect of LSEC from ImmTOR-treated animals was dose-dependent and of much higher scale (by the factor of 3 to 10) and was confirmed by two independent methods of proliferation measurement (**Figures 7A, B**). When cytokine expression by Kupffer cells and LSEC isolated from ImmTOR-treated mice was assessed *in vitro vs.* that in naïve mice, there was a significant decrease in expression of KC/GRO (**Figure 7C**), a neutrophil chemokine with a known role in several models of inflammatory response (24, 25).

**Figure 8:**
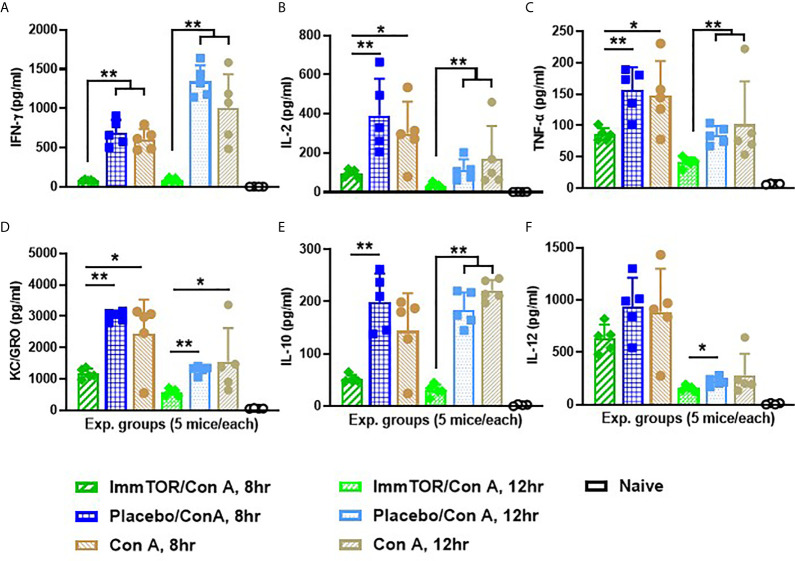
ImmTOR pretreatment diminishes systemic inflammatory cytokines after Con A challenge. Six groups of nice (5 each) which were either untreated or injected with ImmTOR or NP-Empty particles (300 µg) were challenged with concanavalin A (12 mg/kg, i.v.) 7 days after the treatment, sacrificed 8 or 12 hours later and levels of serum cytokines determined. **(A–F)** – serum levels of IFNγ, IL-2, TNFα, KC/GRO, IL-10, and IL-12 correspondingly, are shown for both post-challenge time-points for all experimental groups vs. that of naïve (no ImmTOR, no Con A) mice with statistical significance indicated (* – p<0.05, ** – p<0.01; Mann-Whitney test). Experiment was performed twice with essentially the same outcomes and the results of representative study are shown.

**Figure 9:**
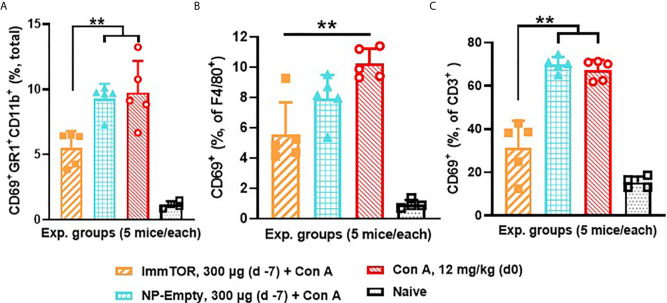
ImmTOR pretreatment diminishes hepatic neutrophil infiltration as well as hepatic macrophage and T cell activation after concanavalin A challenge. Three groups of mice (5/group) which were either untreated or pre-injected with ImmTOR or NP-Empty particles (300 µg) were challenged with concanavalin A (12 mg/kg, i.v.) 7 days after the treatment, sacrificed 12 hours later, livers processed to single cell suspensions and analyzed by FACS. **(A)** – fraction of activated neutrophils (identified as CD69^+^GR1^+^CD11b^+^) out of total hepatic cells; **(B, C)** – fractions of activated (CD69^+^) macrophages and T cells (identified as F4/80^+^ and CD3^+^ cells, correspondingly) are shown *vs.* that of naïve (no ImmTOR, no Con A) mice with statistical difference between groups indicated (** – p<0.01; Mann-Whitney test).

**Figure 10:**
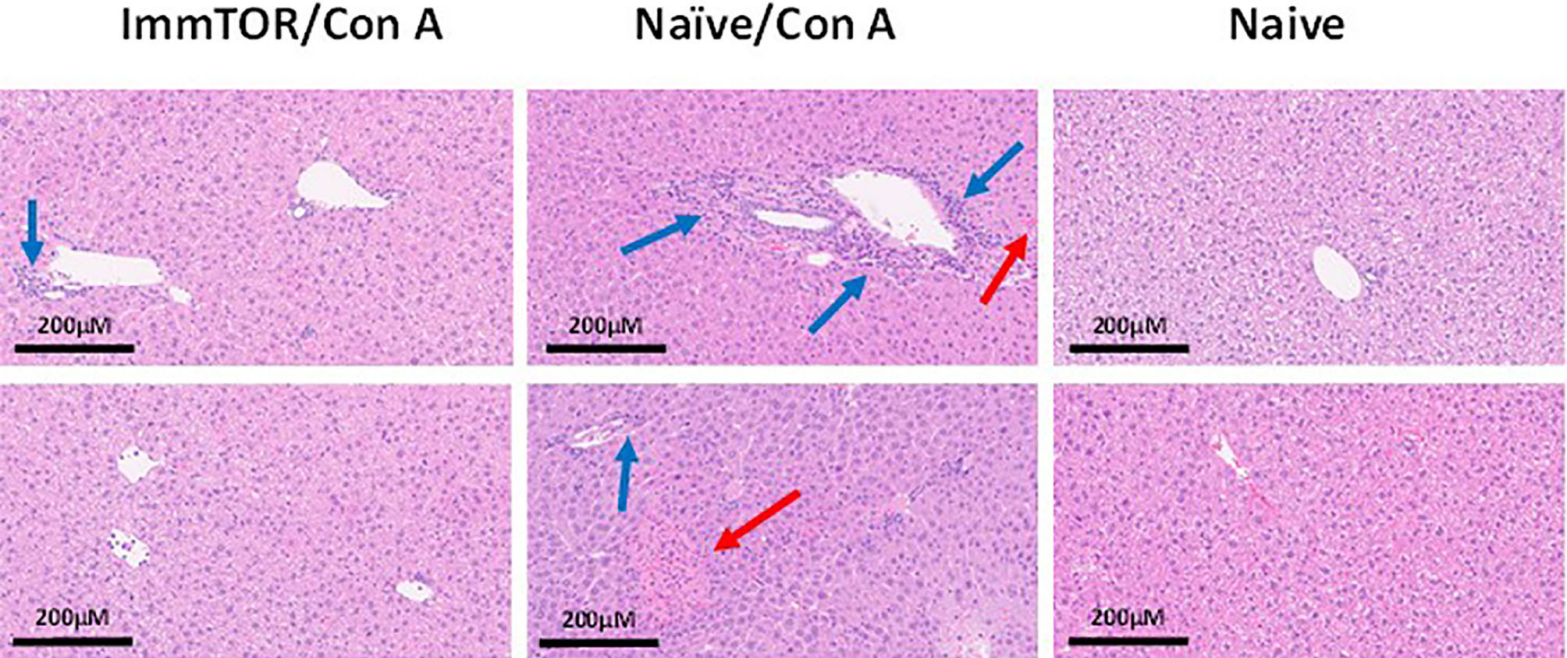
ImmTOR pretreatment diminishes hepatic cytotoxicity after Con A challenge. Mice which were either untreated or pre-injected with ImmTOR particles (300 µg) were challenged with concanavalin A (12 mg/kg, i.v.), sacrificed 24 hours later, livers fixed and processed to tissue slides, stained by hematoxylin-eosin, and analyzed microscopically. The areas of leukocyte infiltration adjacent to vessels (blue arrows) and necrotic areas (red arrows) are indicated. Samples from two representative animals are shown for both groups *vs.* naïve controls (no treatment, no Con A) as indicated.

Since neutrophils along with several other immune cell types are known to play a key role in a model of immune-mediated hepatic cell damage inflicted by concanavalin A (Con A) administration (20, 21, 26), we have then tested the ability of ImmTOR to prevent Con A-mediated liver inflammation. When mice treated with ImmTOR at 7 days prior to Con A challenge were evaluated, there was massive decrease in systemic cytokine induction *vs.* that seen in untreated mice or those treated with NP-Empty placebo, with the difference in levels of IFN-γ, KC/GRO, TNFα, IL-2 and IL-10 being especially pronounced at several post-challenge time-points tested (**Figure 8**). Similarly, intrahepatic appearance of activated neutrophils as well as activation of liver-resident macrophages and T cells was significantly suppressed by ImmTOR, but not by NP-Empty pre-treatment (**Figure 9**) as was histological evidence of leukocyte infiltration of blood vessel-adjacent liver tissues and Con A-induced tissue necrosis (**Figure 10**). Similar effect, but of lesser scale was seen if ImmTOR treatment was administered 3 days prior to Con A challenge (not shown).

## Discussion

The liver is a unique organ that receives eighty percent of its blood supply from the hepatic portal vein, which carries a massive load of foreign antigens such as harmless dietary antigens as well as commensal bacterial degradation products. Unbridled immune responses against such antigens would be detrimental (27–29). Thus, the liver functions as an important immune organ that has evolved redundant pathways to maintain immunological tolerance to harmless antigens, while retaining the capacity to respond to potentially dangerous pathogens. In the current study, we demonstrate that biodegradable ImmTOR nanoparticles encapsulating rapamycin are taken up by all major resident liver cell populations evaluated, including KC, LSEC, hepatocytes, HSC and DC, and enhance the tolerogenic microenvironment in the liver by modulating surface expression of co-stimulatory molecules and checkpoint molecules on antigen presenting cells in the liver, increasing the percentage of hepatic T cells with a regulatory phenotype, elevating PD-1 expression on T cells, and by dramatically downregulating expression of CD4 and CD8 on effector T cells, leading to the emergence of a large population of DN T cells.

The liver contains multiple types of cells that are capable of presenting antigen to T cells. The KC are the most abundant liver resident macrophage population possessing scavenger/phagocytic function (15), allowing them to bind and take up many endogenous and foreign molecules (30, 31). Several KC subpopulations have been described, depending on their cytokine-secreting and phagocytic abilities (32). KC express high levels of MHC class II and co-stimulatory molecules, which support effector T cell responses; however, antigen presentation by KC cells can result in induction of Tregs and immune tolerance, if expression of PD-L1 or other immunosuppressive signaling molecules are upregulated (33–37). ImmTOR was taken up by the vast majority of KC cells of either cytokine-secreting (F4/80^+^, CD11b^+^) or phagocytic (F4/80^+^, CD68^+^) phenotype (**Figures 1A, B**) and induced a tolerogenic phenotype in KCs, as evidenced by substantial increase in PD-L1 expression and decrease in CD80 and MHC class II expression that persisted for 7 days after treatment (**Figures 2A–C**). Dendritic cells showed a similar response to ImmTOR, although to a lesser degree than KCs.

The process of maintaining immune tolerance in the liver is not confined to professional APC, such as KC, but is known to be multifaceted with hepatocytes, stellate cells and especially LSEC playing a key role in this process. LSECs line the liver sinusoids and exhibit extraordinary scavenger function. LSECs are the most efficient endocytic cell population in the body and are involved in scavenging molecules from the bloodstream (38). LSECs act as sentinel cells that can detect microbial pathogens in the blood through pattern recognition receptors, and express MHC and costimulatory molecules to support antigen-presentation to CD8^+^ and CD4^+^ T cells (39–41). The tolerance-inducing properties of LSEC have been closely linked to their PD-L1 expression during antigen presentation to T cells (27, 42) leading to induction or activation of Treg cells (43). ImmTOR treatment induced downregulation of immunostimulatory molecules and MHC class II molecules and upregulation of PD-L1, which was especially pronounced and prolonged (up to two weeks). Other parenchymal cells, such as hepatocytes and stellate cells, showed a similar response, although the effects in those were less pronounced and limited to those cells that took up ImmTOR. Thus, different hepatic cell populations are differentially affected by ImmTOR due to differences in ImmTOR uptake and resulting phenotype.

T cell populations also exhibited profound and varied phenotypic changes in response to ImmTOR treatment. We observed a marked increase in T cells with a regulatory phenotype (CD25^+^, CD127^low^, CD4^+^, CD3^+^, (**Figures 5F** and **6C**). ImmTOR also induced broad PD-1 elevation across both CD4^+^ and CD8^+^ T cells as well as on CD4 T cells with a regulatory phenotype (**Figure 5**). The increase in PD-1 expressing T cells was inhibited by antibodies against PD-L1 and CTLA-4 (**Figure 6**).

In addition to T cells with a phenotype similar to classic Tregs, there was an emergence of CD3^+^ T cell population lacking expression of CD4 and CD8 (CD3^+^CD4^–^CD8^–^ or double-negative, DN T cells) (**Figures 4**). This effect was not observed in splenic T cells, despite the fact that ImmTOR traffics to the spleen as well as to the liver (7). Double negative T cells have been shown to exhibit tolerogenic properties and have the ability to strongly suppress activated CD8^+^ and CD4^+^ T cells and impair their metabolism (44–48). Consistent with these observations, DN T cells are known to play a role in suppressing the immune response in transplantation (49) and graft versus host (GvHD) disease, with severity of GVHD in recipients of allogeneic hematopoietic stem cells being in inversely correlated with the number of circulating DN T cells (50). Interestingly, the effect of ImmTOR on the emergence of DN T cells was not dependent on the PD-L1/PD-1 axis or CTLA-4, in contrast to the effect of ImmTOR on PD-1 expression (**Figure 5**) and CD25^+^, CD127^low^, CD4^+^, CD3^+^ T cells. Conversely, there have been reports of DN T cells exhibiting inflammatory functions in a number of autoimmune diseases (reviewed in 51), but these seem to be associated with systemically active DN T cells as opposed to those induced locally with no similar data with respect to liver-specific DN T cells.

We sought to overcome an initial limitation of this research, which confined it to studying phenotypic changes in liver cell populations by conducting functional studies ex vivo. Indeed, LSEC from ImmTOR-treated animals were capable of suppressing proliferation of stimulated T cells and they (as well as KC) also expressed much lower amounts of neutrophil chemokine KC/GRO (**Figure 7**), which is known to play a key role in several models of inflammatory response (24, 25, 52) and hepatic production of which has been shown to correlate with hepatic infiltration by neutrophils in a model of experimental sepsis (53). There is also some evidence of KC/GRO directly affecting T cells leading to preferential naïve CD4 T-cell differentiation to Th17 (52).

Therefore, it was not surprising that hepatic infiltration by activated neutrophils as well as activation of liver-resident macrophages and T cells after Con A challenge, typical features of this model of immune-mediated liver toxicity (20, 21, 26), were diminished by ImmTOR pretreatment (**Figure 9**). This was also true for other hallmarks of Con A-induced liver pathology such as leukocyte-induced cell death and systemic cytokine induction (**Figures 8** and **10**). Some of these effects have been observed earlier in a small study using free rapamycin which was systemically administered shortly before Con A challenge (54). However, the action of ImmTOR has a much broader window of efficacy and seems to affect many other cytokines in addition to those earlier reported (54), especially interesting being those, which were not seen previously as significant actors in this system, specifically KC/GRO.

Previous studies have demonstrated that ImmTOR is capable of inducing durable immune tolerance *in vivo* to model antigens, such as ovalbumin and KLH, against a broad range of biologic therapies, and to autoantigens (2–8, 55). As reported earlier, ImmTOR particles are 150 nm in diameter and have a surface charge of 8.9 ± 0.1 mV (55) with rapamycin load within 8-25% weight and release rate up to 60% at 1 hour or 25-80% at 24 hours. A role for the spleen in the mechanism of action of ImmTOR was inferred by the appearance of splenic antigen-specific regulatory T cells; however, whether those Tregs arose in the spleen or migrated there was not addressed (2, 6, 7). Splenectomy substantially, but incompletely, negated the tolerogenic effects of ImmTOR (2). However, there was no major effect of ImmTOR on dendritic cell phenotype, including MHC class II and co-stimulatory molecule expression, or total T cell sub-populations in the spleen (2). Indeed, the phenotypic changes induced by ImmTOR are considerably more pronounced in the liver than the spleen. In particular, the emergence of a large population of DN T cells was observed in the liver but not the spleen (**Figure 4E**). Future research will focus on developing genetically modified mouse models to further dissect the role of the liver in immune tolerance induction induced by ImmTOR.

While the liver environment favors the induction and maintenance of immune tolerance, tolerance can be broken leading to liver specific autoimmune diseases, such as autoimmune hepatitis, primary biliary cholangitis, and primary sclerosing cholangitis. The biodistribution of ImmTOR nanoparticles to the liver and its multipronged effects on promoting a tolerogenic phenotype in antigen-presenting cells and T cells suggest that ImmTOR may be beneficial in the treatment of liver autoimmune diseases, either alone or combined with autoantigens, such as PDC-E2, to restore immune tolerance to autoantigens.

## Data Availability Statement

The original contributions presented in the study are included in the article/**Supplementary Material**. Further inquiries can be directed to the corresponding author.

## Ethics Statement

The animal study was reviewed and approved by Institutional Animal Care and Use Committee (IACUC) of Selecta Biosciences.

## Author Contributions

PI, CR and GR designed the experiments, CR, JL and GR executed the studies, PI, CR, JL, GR and TK analyzed the data, PI and TK wrote the manuscript. All authors contributed to the article and approved the submitted version.

## Funding

This work was funded by Selecta Biosciences. The funder was not involved in the study design, collection, analysis, interpretation of data, the writing of this article or the decision to submit it for publication.

## Conflict of Interest

All authors are employees and shareholders in Selecta Biosciences, Inc
