# Supplementary material for: Enhancement of the Tolerogenic Phenotype in the Liver by ImmTOR Nanoparticles
Source: Front Immunol. 2021 May 25;12:637469. doi: 10.3389/fimmu.2021.637469 (PMC8186318; doi:10.3389/fimmu.2021.637469)
Supplement: Supplementary file 1 [file DataSheet_1.docx]

Undesirable immune responses can compromise the efficacy or safety of biological therapies or cause autoimmune diseases. Recently, biodegradable nanoparticles, called ImmTOR, have been shown to induce durable tolerogenic immune response to co-administered biologics and autoantigens. Here we demonstrate the ability of ImmTOR to enhance the natural tolerogenic environment of the liver. Following intravenous administration, all major populations of liver cells took up fluorescent-labeled ImmTOR particles, which resulted in downregulation of co-stimulatory molecules associated with immune stimulation and upregulation of the checkpoint molecules involved in immune tolerance. A specialized endothelial cell population, called Liver Sinusoidal Endothelial Cells, emerged as a key target cell type for ImmTOR. The tolerogenic environment led to a multi-pronged modulation of hepatic T cell populations, including an increase in T cells with a regulatory phenotype and the emergence of a large population of T cells lacking expression of CD4 and CD8 differentiation markers. These results suggest that the liver may contribute to the tolerogenic properties of ImmTOR treatment, and that ImmTOR may be a potential therapy for liver autoimmune diseases.
