# Supplementary material for: Enhancement of the Tolerogenic Phenotype in the Liver by ImmTOR Nanoparticles
Source: Front Immunol. 2021 May 25;12:637469. doi: 10.3389/fimmu.2021.637469 (PMC8186318; doi:10.3389/fimmu.2021.637469)
Supplement: Supplementary file 2 [file DataSheet_2.pdf]

Supplementary Material

1 Supplementary Figures

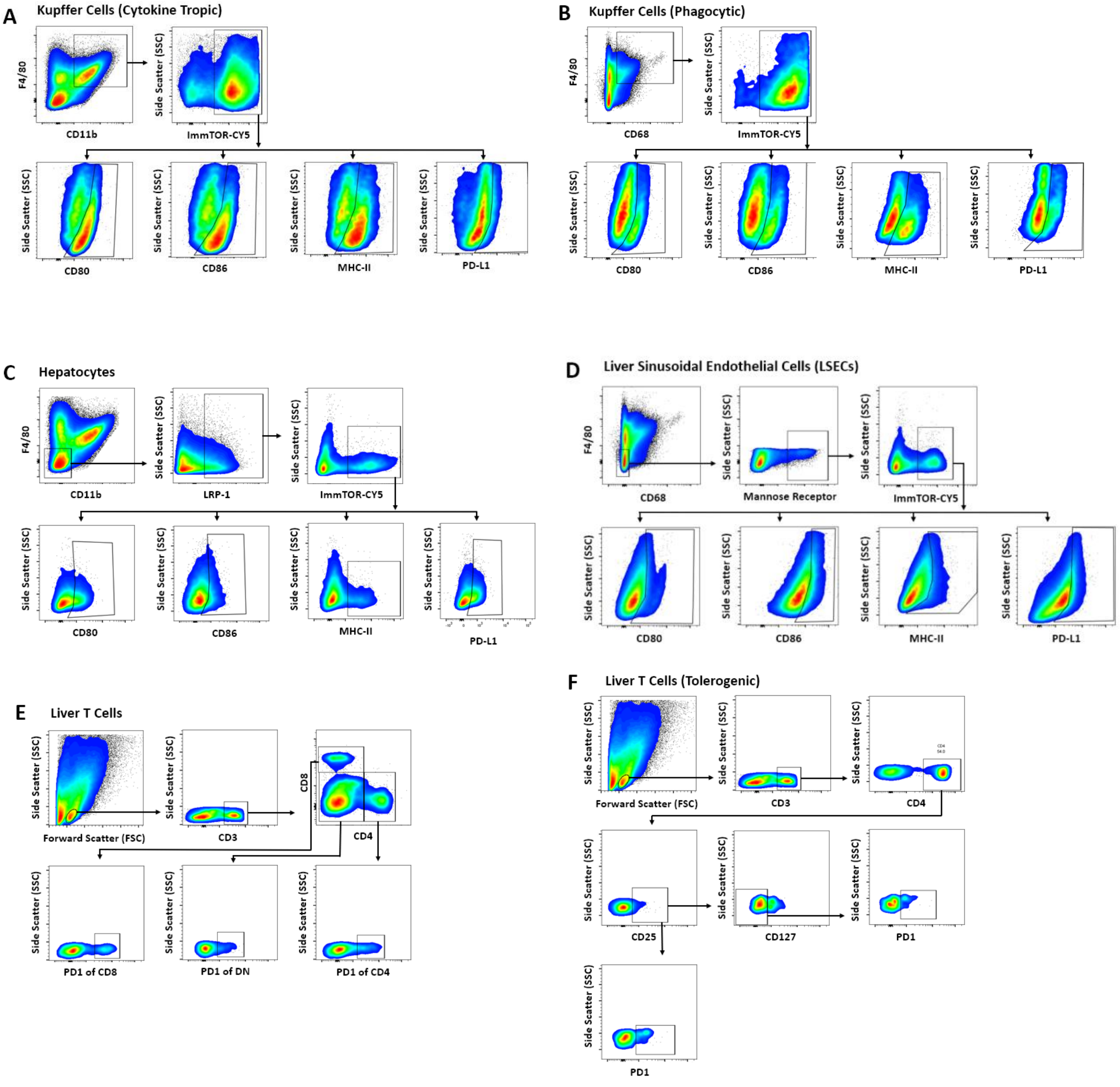

Supplementary Figure S1. Flow cytometry gating strategy employed for the identification of hepatic cell populations. **A-D**. Kupffer cells (cytokine-tropic and phagocytic, respectively), hepatocytes and liver endothelial sinusoidal cell (LSEC) identification, including labeled nanoparticle (ImmTOR) uptake and activation state. **E**. Liver resident T cell identification, including $CD4^+$, $CD8^+$ and double-negative (DN) T Cells characterization and their activation state. **F**. Liver resident tolerogenic T cell identification and their activation state. For all samples blinded reproduction of gates was based on initial independent control sample(s).

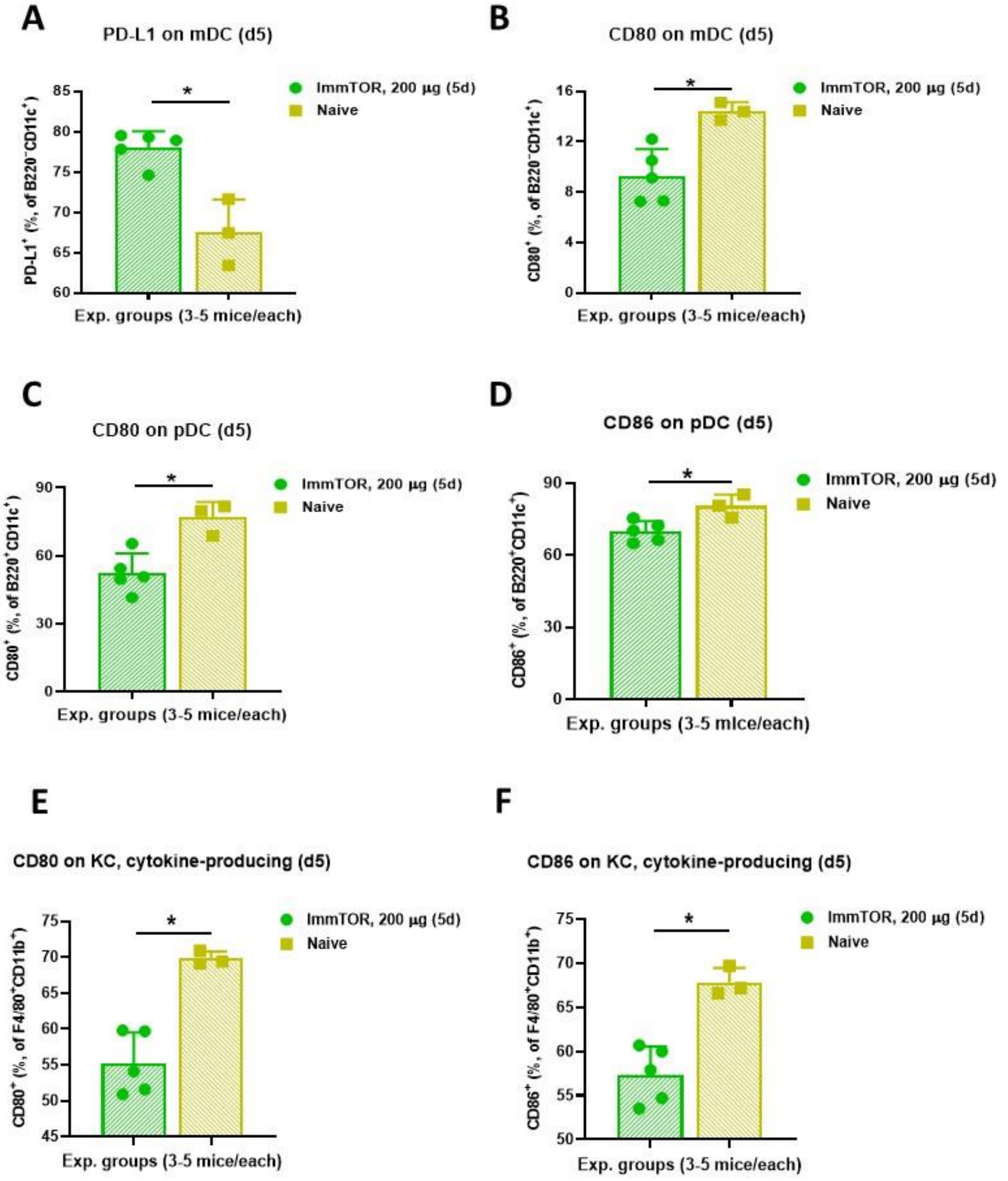

Supplementary Figure S2. Effect of ImmTOR on hepatic professional antigen-presenting cells. Livers were processed at five days after ImmTOR injection, stained and analyzed by flow cytometry. Fractions of PD-L1^{+} (A) and CD80^{+} (B) myeloid DC (mDC, identified as B220^{-}CD11c^{+}) and CD80^{+} (C, E) and CD86^{+} (D, F) plasmacytoid DC (C, D; pDC, identified as B220^{+}CD11c^{+}) and cytokine-producing KC (E, F; identified as F4/80^{+}CD11b^{+}) are shown. Statistical difference in the size of respective fractions vs. that in naïve mice is shown (* – $p<0.05$; Mann-Whitney test).

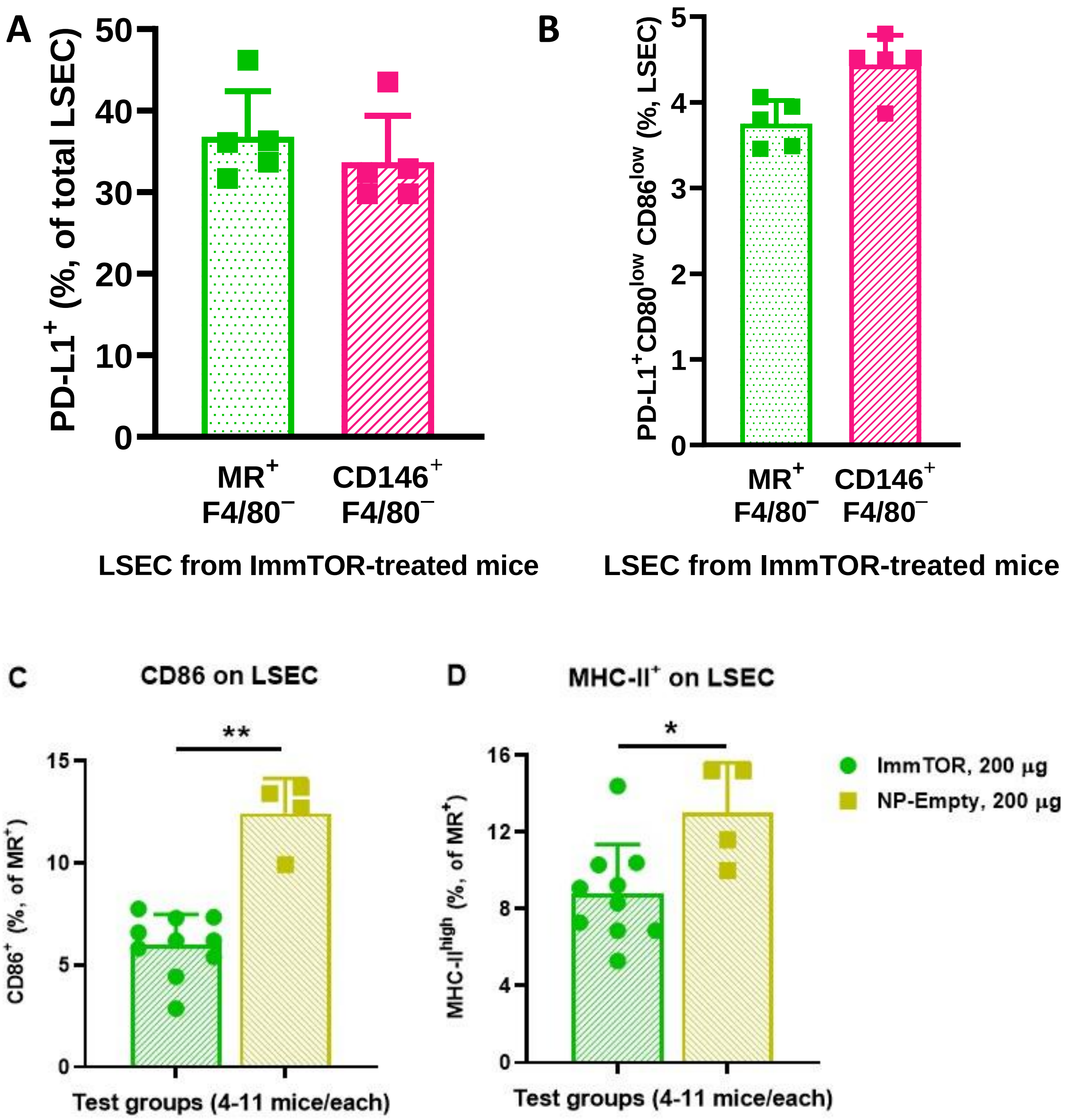

Supplementary Figure S3. Effect of ImmTOR on liver sinusoidal endothelial cells (LSEC) is phenotypically indistinguishable whether MR or CD146 is used for LSEC surface staining and cannot be replicated by NP-Empty. Livers were processed at five (A, B) or seven (C, D) days after ImmTOR injection, stained and analyzed by flow cytometry. Fractions of PD-L1$^+$ (A) or PD-L1$^+$ CD80^{low} CD86^{low} (B) LSEC, identified either as MR$^+$ F4/80$^-$ (green) or as CD146$^+$ F4/80$^-$ (purple) are shown (A, B) as are fractions of CD86$^+$ (C) and MHC-II$^+$ (D) LSEC identified as MR$^+$ (C, D). Statistical difference in the size of respective fractions is shown (* – $p<0.05$; Mann-Whitney test).

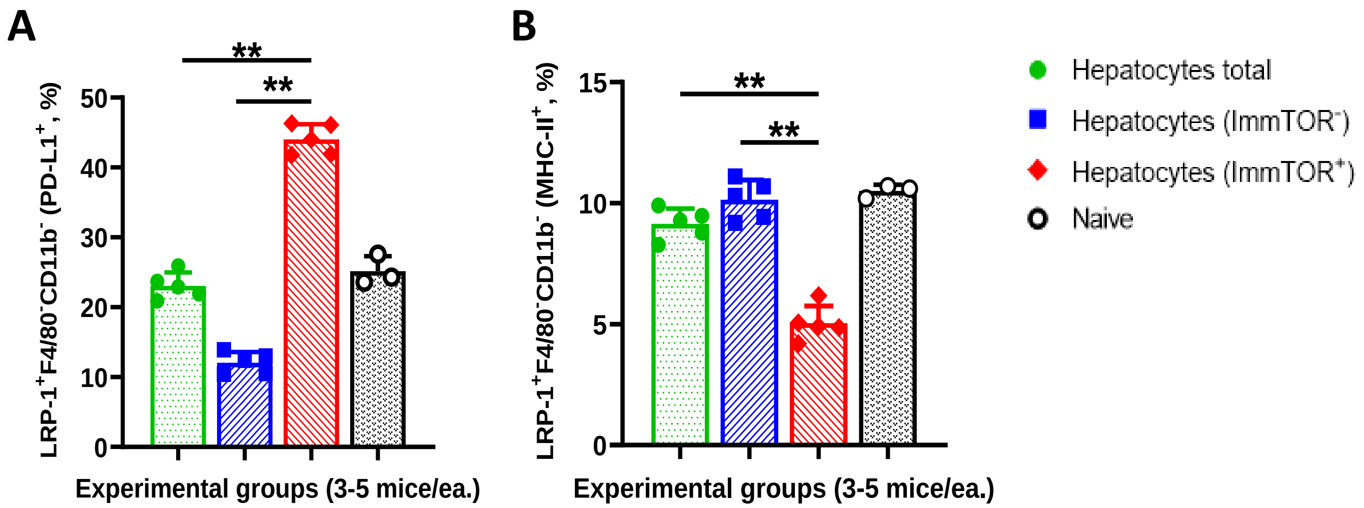

Supplementary Figure S4. ImmTOR effects on PD-L1 and MHC class II surface expression in hepatocytes. Seven days after i.v. injection of ImmTOR-Cy5 (200 µg), livers were processed, stained and analyzed by flow cytometry. Fractions of PD-L1$^+$ (A) and MHC-II$^+$ (B) hepatocytes (identified as LRP-1$^+$F4/80$^-$CD11b$^-$) are shown for total hepatocytes (green bar), ImmTOR-negative cells (blue bar) and ImmTOR-positive cells (red bar). Statistically significant difference in the size of respective fractions is shown (** – $p<0.01$; Mann-Whitney test).

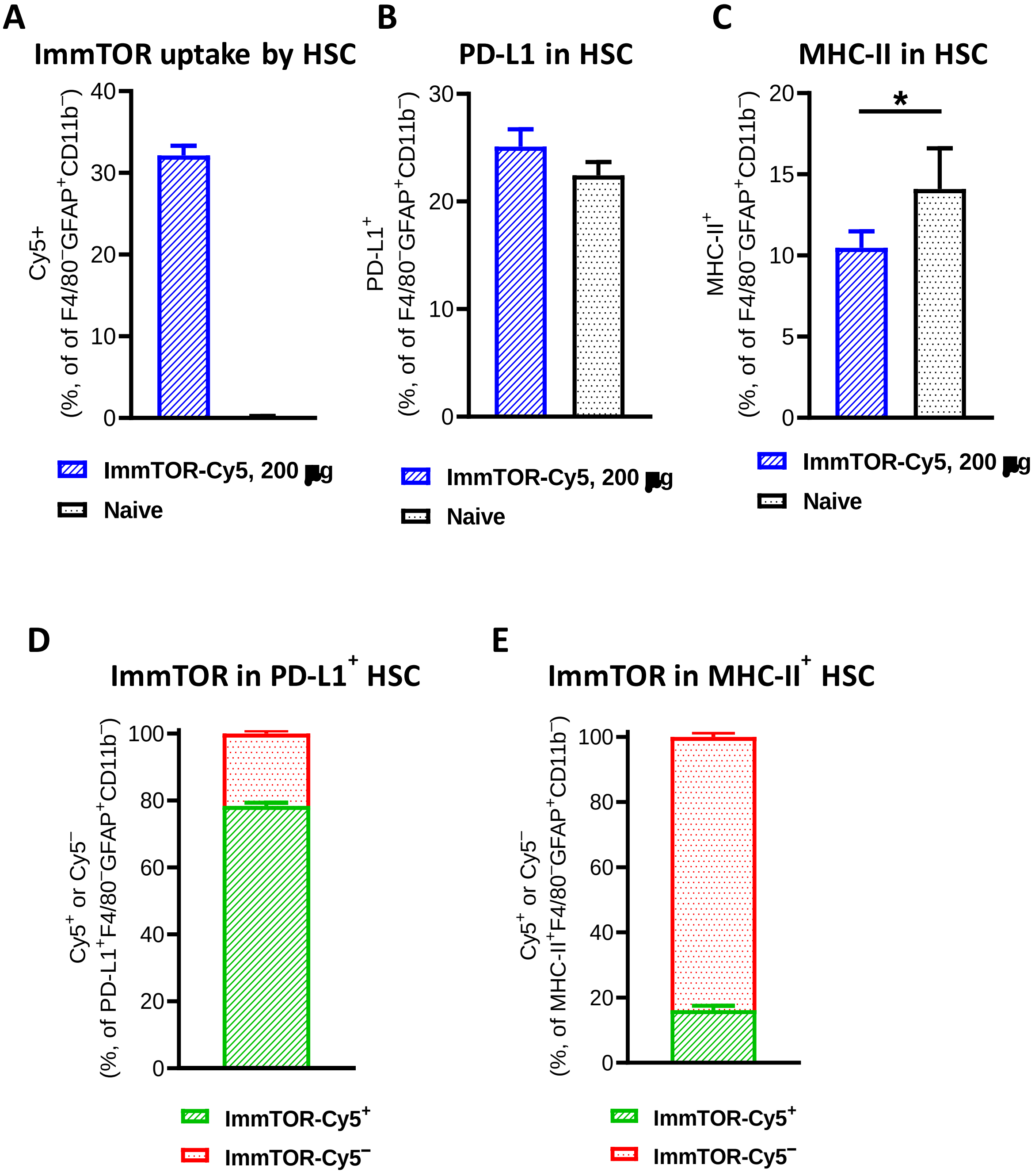

Supplementary Figure S5. Uptake of ImmTOR and its effects on hepatic stellate cells (HSC). HSC were analyzed at five days after injection of ImmTOR-Cy5 (200 µg; 5 mice/group). Fractions of ImmTOR-containing (A) HSC (identified as $F4/80^{-}GFAP^{+}CD11b^{-}$) followed by fractions of PD-L1^{+} (B) and MHC-II^{+} (C) HSC are shown. Relative proportion of ImmTOR-positive and ImmTOR-negative cells in PD-L1^{+} (D) and MHC-II^{+} (E) is also shown. Statistical difference in the size of respective fractions vs. that in naïve mice is shown (* – $p<0.05$; Mann-Whitney test).

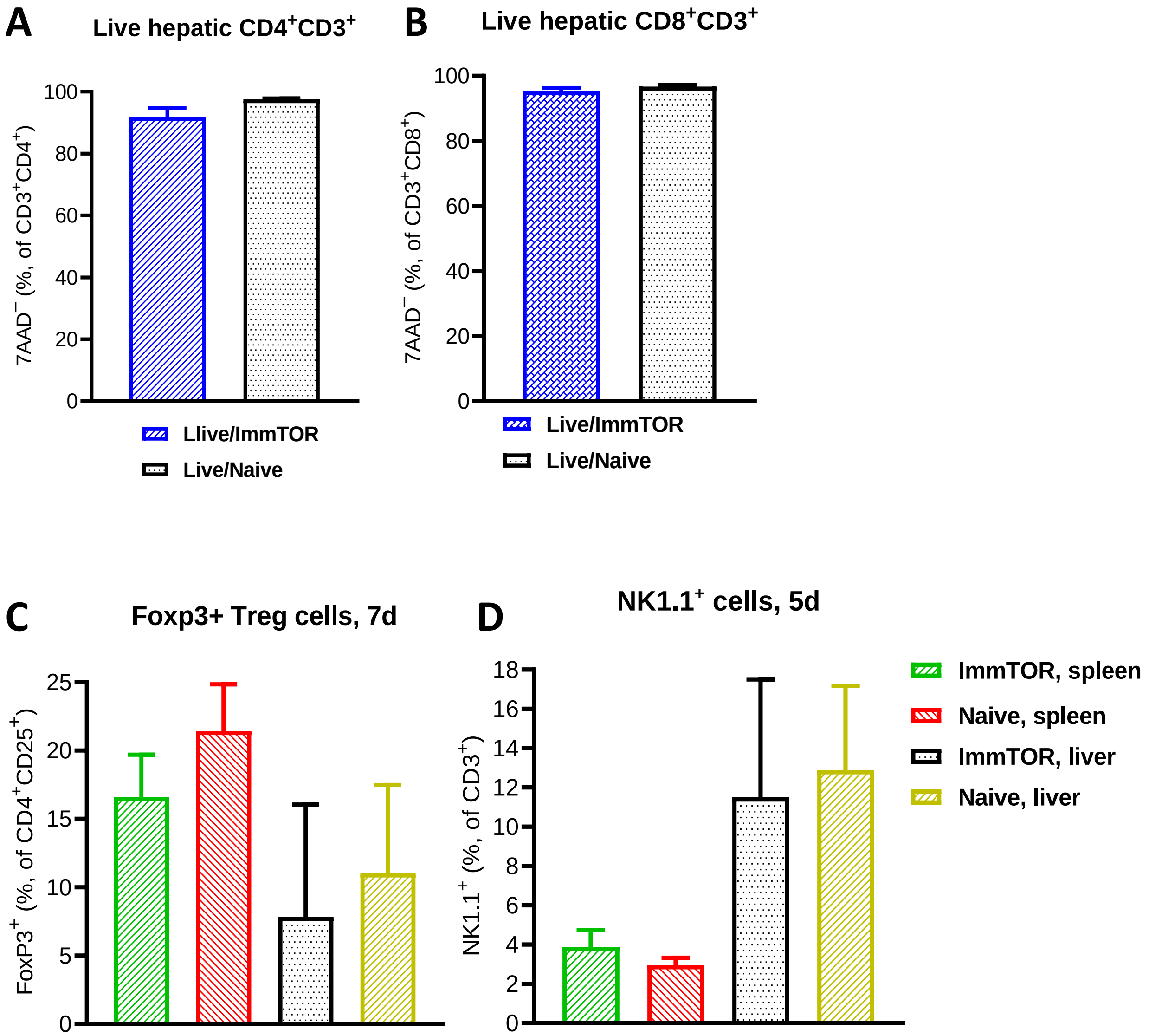

Supplementary Figure S6. ImmTOR does not affect CD4$^+$ and CD8$^+$ T cell viability, induction of FoxP expression or NK T cells. Five or seven days after i.v. injection of ImmTOR at 200 µg livers (A, B) or spleens and livers (C, D) were taken, stained with antibodies to surface markers and additionally either with 7AAD, which is excluded by living cells (A, B) or permeabilized and then stained with antibody to FoxP3 (C) and analyzed by flow cytometry. Fractions of hepatic 7AAD$^-$ CD3$^+$CD4$^+$ (A) or CD3$^+$CD8$^+$ (B) CD4$^+$CD25$^+$CD127^{low} regulatory cells are shown (n=6 mice/group). C, D. Fractions of splenic and hepatic FoxP3$^+$CD4$^+$CD25$^+$ (C) or of NK1.1$^+$ CD3$^+$ cells are shown (n=3-6 or 6-12 mice/group, respectively). Results of representative studies repeated at least twice are presented.

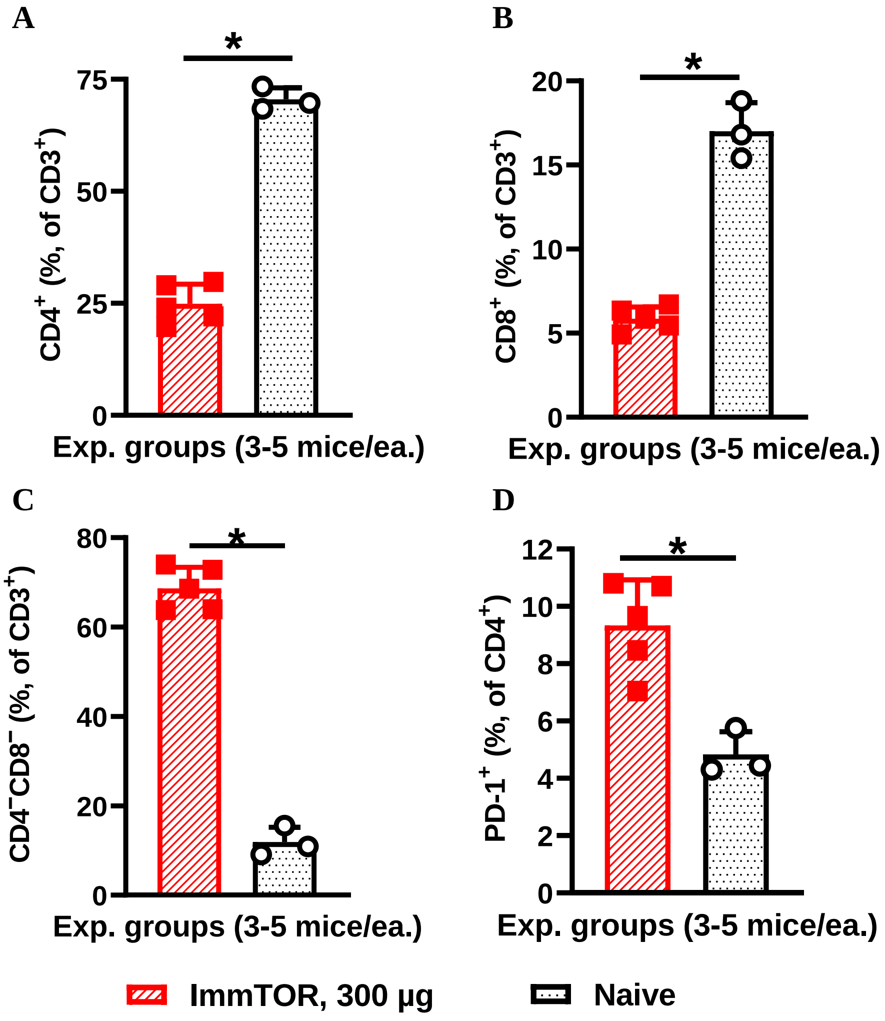

Supplementary Figure S7. ImmTOR leads to emergence of double-negative hepatic T cells and PD-1 upregulation in male mice. Male BALB/c mice (5/group) were injected with ImmTOR (i.v., 300 µg) and 14 days later livers were processed, stained with antibodies to surface markers and analyzed by FACS. Fractions of 1 $CD4^+$ (A), $CD8^+$ (B), $CD4^-CD8^-$ $CD3^+$ (C) and PD-1+$CD4^+$ (D) T cells are shown vs. that in naïve mice with statistical significance indicated (* – $p<0.05$; Mann-Whitney test).
